# Significance of surgical first assistant expertise for surgical site infection prevention: Propensity score matching analysis

**DOI:** 10.1097/MD.0000000000033518

**Published:** 2023-04-14

**Authors:** Deok Ryeong Kim, Byul Hee Yoon, Yung Ki Park, Byung Gwan Moon

**Affiliations:** a Department of Neurosurgery, Uijeongbu Eulji Medical Center, Eulji University, Seoul, Korea.

**Keywords:** laminectomy, physician assistant, propensity score, surgical site infection

## Abstract

Surgical site infection (SSI) is one of the most common postoperative complications in patients undergoing major operations, such as spinal fusion surgery, and a major contributor to patient morbidity and mortality. SSI is considered the most preventable type of infection; however, the risk of SSI is multifactorial. This study aimed to determine the extent to which the expertise of the surgical first assistant (SFA) affected SSI rates. We retrospectively reviewed 528 patients at a single institution who underwent lumbar spine fusion surgery via the posterior approach performed by a single surgeon between January 2012 and May 2020. The SFAs participating in the surgeries were classified into 2 groups: a certified neurosurgery specialist and relatively less experienced neurosurgery resident trainees. To reduce potential selection bias and confounding factors, propensity score matching was performed between the 2 groups. In 170 of the 528 lumbar spine fusion surgeries, the SFA was a certified neurosurgery specialist. In the other 358 surgeries, the SFA was a resident trainee. Seventeen patients met the SSI criteria. The SSI rate was significantly different between the 2 groups (0.6% (1 patient) and 4.5% (16 patients) in the certified specialist and resident trainee groups, respectively; *P* = .02). After propensity score matching, 170 paired patients were selected. After adjusting for confounding factors, SFAs that were certified neurosurgery specialists were associated with a lower likelihood of SSI (adjusted OR 0.09; 95% CI, 0.01 to 0.79; *P* = .029) than SFAs that were neurosurgery residents. A higher level of SFA expertise was significantly associated with a lower overall SSI rate in lumbar spine fusion surgeries. It is difficult to predict the incidence of SSI; however, this finding suggests the importance of SFA expertise in preventing SSI.

## 1. Introduction

Surgical site infection (SSI) is a serious postoperative complication.^[1]^ According to the centers for disease control (CDC) and prevention, approximately 500,000 SSIs occur each year, accounting for almost 1-quarter of all annual nosocomial infections in the United States.^[2]^ SSI reduces the health-related quality of life, doubles the risk of rehospitalization, extends hospitalization, and increases hospital costs.^[2]^ Understanding and mitigating SSI risk factors are necessary to reduce the risk of SSI.^[3]^ Various authors have identified several SSI risk factors, which are divided into 2 categories: patient-related risk factors and procedure-related risk factors.^[4–6]^ Procedure-related risk factors, over which the surgeons have more direct control, include the number of people in the operating room, screening and decolonization programs, perioperative skin preparation, hand hygiene, and factors related to proficiency, including the duration of the surgical procedure.^[7]^ Regarding surgical experience, several studies investigating the impact of expertise on surgical outcomes have drawn similar conclusions: surgeons performing specialized operations in large quantities have better patient outcomes than surgeons performing fewer operations.^[8–12]^ These claims indicate the importance of the surgical first assistant (SFA), an essential member of the surgical team. In the operating room, the team collaborates to ensure that the patient receives optimal care during surgery. The SFA works closely with the surgeon to facilitate the surgical technique and workflow. With regard to the effect on SSI risk, it is believed that the expertise of the SFA is as important as that of the operating surgeon, but this hypothesis has not yet been investigated. In this study, we investigated relevant factors that affect the risk of SSI and analyzed the importance of SFA expertise in reducing SSI rates following spine fusion surgery. These findings may also be applicable to other surgical specialties.

## 2. Methods

### 2.1. Patients population

Following administrative approval from the institutional review board, we retrospectively reviewed 528 patients who underwent lumbar spine fusion surgery via the posterior approach at a single institution between January 2012 and May 2020. All surgical procedures for patients included in this study were performed by only 1 neurosurgeon (BG Moon). Patients who underwent a revision spinal surgery or who had documentation suggestive of current infection at the time of initial admission were excluded. We used the CDC definitions of nosocomial SSIs to identify SSIs within the group of 528 patients who met the inclusion criteria for our study. The CDC classifies SSIs as incisional or organ/space. Incisional SSIs are subclassified into those involving only the skin and subcutaneous tissue (superficial incisional SSI) and those involving the deep soft tissues of the incision (deep incisional SSI). Organ/space SSIs involve any part of the anatomy other than the incision that was opened or manipulated during the operative procedure.^[13]^ The medical records of the patients were reviewed to confirm the presence of an SSI, as defined by the CDC guidelines.^[14]^ According to these guidelines, 17 patients had a confirmed SSI following posterior lumbar arthrodesis with instrumentation. We hypothesized that one of the major modifiable risk factors for SSIs in spinal surgeries was the use of a relatively less trained surgical resident as the SFA. To verify this hypothesis, the SFAs included in this study were classified into 2 groups. In the first group, surgeries included a single SFA, who was a certified neurosurgery specialist with more than 10 years of experience. In the second group, surgeries included several SFAs, who were relatively less experienced neurosurgery resident trainees.

### 2.2. Infection control procedure

During the study period, all surgery and infection control procedures remained almost unchanged. Surgical site preparation was performed using an antiseptic combination of alcohol and iodophors, which provide strong, immediate action and persistent activity, respectively. In almost every case, only 1 neurosurgeon performed the posterior instrumented lumbar arthrodesis operation. In accordance with standard practice, antibiotic prophylaxis was administered using a first-generation cephalosporin based on the patient’s weight, starting at least 1 hour before the skin incision and continuing for 24 hours postoperatively.^[15]^

### 2.3. Data collection

Preoperative patient characteristics, including age, sex, medical comorbidities, body mass index (BMI), and smoking history, were recorded. Investigators collected perioperative data including laboratory values, microbiological information, number of levels fused, and operative time. Other investigators performed comprehensive logic checks of these data to identify illogical or impossible data. All illogical data were examined several times by comparing medical and electronic patient records.

### 2.4. Statistics

Data are shown as the number (%) for categorical variables and the mean for continuous variables. To compare the characteristics of the study groups, the chi-square test was used for categorical variables, and Student *t* test was used for continuous variables. Multivariate logistic regression was used to evaluate the independent associations of each potential explanatory variable. All variables previously identified in the literature were considered eligible for inclusion in the model. Propensity score matching was used to control for potential confounding variables and reduce the number of disparities between the 2 groups. The 2 groups of patients were matched 1-to-1 by nearest-neighbor matching with a caliper distance of 0.05. Variables affecting SSI outcomes were selected, including age, sex, diabetes mellitus, BMI, smoking, and hyperlipidemia. Propensity score matching was conducted using the MatchIt package (version 2.4-21) in R (R for Windows 3.3.4; The R Foundation for Statistical Computing, Vienna, Austria). Statistical analyses were performed using IBM SPSS Statistics version 18.0.0 for Windows (SPSS Inc, Chicago, IL).

### 2.5. Ethics statement

This study was approved by the Institutional Review Board of Eulji University Hospital and the requirement for informed consent was waived (IRB No. 2018-08-003).

## 3. Results

### 3.1. Characteristics of the study population

In total, 528 patients met our inclusion criteria. A total of 17 cases (3.2%) met the CDC criteria for SSI, an overall infection rate similar to that reported in the literature.^[16,17]^ At surgery, the mean age was 66 ± 9.9 years (range, 37–88 years), and 194 patients were male (37%). Patients were divided into 2 groups according to the expertise of the SFA. In group 1, the SFA was a certified neurosurgery specialist with more than 10 years of experience, and in group 2, the SFAs were relatively less experienced resident trainees. The preoperative patient characteristics and comorbidities of the groups are shown in Table 1. Operative time and SSI data are also summarized. There were no significant differences in sex, age, comorbidity, BMI, smoking history, or operative time between the groups, although group 1 included higher percentages of older patients, female patients, and patients with medical comorbidities. However, group 1 patients exhibited a significantly lower risk of SSI than group 2 patients. Of the 17 total cases, only 1 case of SSI was observed in group 1. The infection rates in the neurosurgery resident trainee group and certified neurosurgery specialist group were 4.5% and 0.6%, respectively (*P* = .036).

**Table 1:** Baseline characteristics of the patients.

	Group 1 (n = 170)	Group 2 (n = 358)	*P* value	SMD
Sex (male)	50 (29.4%)	144 (40.2%)	.117	0.228
Age (mean ± SD)	67.4 ± 9.5	65.2 ± 10.0	.087	0.225
Operative time (min)	274.8 ± 87.6	290.4 ± 88.2	.181	0.177
Diabetes mellitus	56 (32.9%)	112 (31.3%)	.898	0.035
Hyperlipidemia	36 (21.2%)	68 (19.0%)	.802	0.054
Smoking	20 (11.8%)	44 (12.3%)	1	0.016
BMI (mean ± SD)	25.4 ± 3.6	27.2 ± 26.2	.387	0.093
SSI	1 (0.6%)	16 (4.5%)	.036*	0.249

BMI = body mass index, SD = standard deviation, SSI = surgical site infection.

### 3.2. Factors predicting SSI

The univariate and multivariate logistic regression models for predicting SSI are shown in Table 2. According to the univariate analysis, the expertise of the SFA was associated with the development of an SSI (*P* = .046). After adjusting for confounding factors previously identified in the literature, such as age, sex, comorbidities, tobacco use, and operative time, more SFA expertise was the only factor associated with a significantly lower risk of postoperative SSI (adjusted odds ratio, 0.11; 95% confidence interval, 0.01–0.89; *P* = .038). The probability of infection was somewhat higher among patients with increased BMI, although this difference was not statistically significant.

**Table 2:** Logistic regression analyses predicting SSI for all 528 patients.

	Crude			Adjusted		
Variables	OR	95% CI	*P* value	OR	95% CI	*P* value
Sex (male)	0.94	0.33–2.61	.898	0.66	0.19–2.27	.513
Age	1	0.95–1.06	.874	1.02	0.96,1.08	.481
Operative time	1	1–1.01	.439	1	0.99–1.01	.835
Diabetes mellitus						
No	0.89	0.3–2.6	.826	0.55	0.15–1.98	.357
Hyperlipidemia						
No	0.87	0.24–3.13	.826	0.86	0.21–3.55	.835
Smoking						
Yes	1.61	0.44–5.94	.474	1.7	0.3–9.54	.545
BMI	1.13	0.98–1.3	.104	1.15	0.98–1.35	.081
Assist						
Group 1	0.13	0.02–0.96	.046	0.11	0.01–0.89	.038*

BMI = body mass index.

* Adjusted by age, sex, operative time, diabetes mellitus, hyperlipidemia, smoking, and BMI.

### 3.3. Results of propensity score matching

To decrease the effects of potential confounding factors, we used 1-to-1 propensity score matching. The clinical and demographic variables included in the analysis were age, sex, comorbidities, tobacco use, and operative time. Overall, 170 matched pairs were established. The standardized mean differences in the clinical characteristics between the groups decreased. Even after propensity score matching (Table 3), there were also significant differences in the SSI rate between the 2 groups (Group 1: 1/170 (0.6%), Group 2: 9/170 (5.3%), *P* = .023). In the multivariate analysis (Table 4), SFA expertise was the only factor significantly associated with postoperative SSI (adjusted odds ratio, 0.09; 95% confidence interval, 0.01–0.79, *P* = .029)

**Table 3:** Baseline characteristics of propensity score-matched pairs.

	Group 1 (n = 170)	Group 2 (n = 170)	*P* value	SMD
Sex (male)	50 (29.4%)	50 (29.4%)	1	<0.001
Age (mean ± SD)	67.4 ± 9.5	65.4 ± 10.9	.195	0.2
Operative time (min)	274.8 ± 87.9	291.4 ± 89.3	.224	0.187
Diabetes mellitus	56 (32.9%)	56 (32.9%)	1	<0.001
Hyperlipidemia	36 (21.2%)	34 (20.0%)	1	<0.001
Smoking	20 (11.8%)	20 (11.8%)	1	<0.001
BMI (mean ± SD)	25.4 ± 3.6	25.5 ± 3.3	.889	0.021
SSI	1 (0.6%)	9 (5.3%)	.023*	0.408

BMI = body mass index, SD = standard deviation, SSI = surgical site infection.

**Table 4:** Logistic regression analyses predicting SSI for propensity score-matched pairs.

	Crude			Adjusted		
Variables	OR	95% CI	*P* value	OR	95% CI	*P* value
Sex (male)	1.03	0.26–4.16	.966	1.2	0.22–6.67	.837
Age	0.99	0.93–1.05	.726	1.01	0.94–1.09	.721
Operative time	1	1–1.01	.23	1	0.99–1.01	.585
Diabetes mellitus						
No	0.49	0.1–2.39	.378	0.46	0.08–2.67	.385
Hyperlipidemia						
No	0.96	0.2–4.75	.962	1.17	0.19–7.23	.864
Smoking						
Yes	0.82	0.1–6.87	.858	0.98	0.09–10.95	.984
BMI	1.16	0.97–1.39	.109	1.2	0.96–1.51	.113
Assist						
Group 1	0.1	0.01–0.81	.031	0.09	0.01–0.79	.029*

BMI = body mass index, SSI = surgical site infection.

* Adjusted by age, sex, operative time, diabetes mellitus, hyperlipidemia, smoking, and BMI.

## 4. Discussion

### 4.1. Importance and incidence of SSI in instrumented spinal fusion surgery

**Figure 1.**
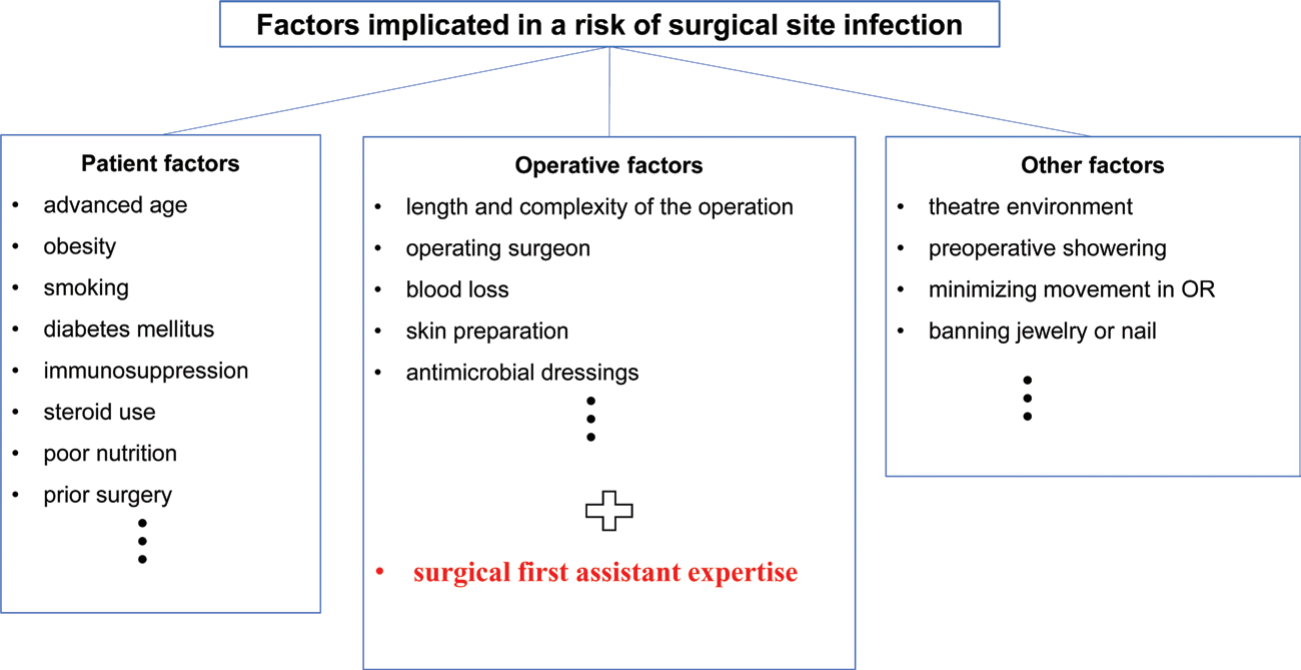
Factors implicated in a risk of surgical site infection.

SSI is a major concern that can lead to significant increases in morbidity and mortality.^[18]^ Moreover, SSI in instrumented spinal fusion surgery is more difficult to treat than that in non-instrumented spinal surgeries and can therefore lead to more serious situations, for the following reasons: The need for long-term antibiotic therapy or reoperation due to exceptionally strong bacterial aggregation and biofilm formation on the indwelling surgical device;^[19]^ The potential need for instrumentation removal and potential risk for nonunion;^[20]^ The potential for progression into central nervous system infection in the event of a cerebral spinal fluid leak, such as a dural tear. Therefore, efforts to reduce SSI rates are urgently needed. Individual institutions have conducted studies on SSI following adult spinal surgery, and infection has been reported to occur in 0.7% to 12.0% of patients.^[3]^ Meanwhile, the National Healthcare Safety Network recorded a mean SSI rate of 0.70% to 4.15% for spinal fusion during 2006 to 2008.^[21]^ The National Nosocomial Infections Surveillance (NNIS) reported an SSI rate of 1.8% following spinal surgery.^[22]^ In our patient population, the overall rate of clinically significant SSIs was 3.2%, which was slightly higher than that in the NNIS report. Infection rate in spinal surgeries can be affected by several factors, including whether fusion is performed. Surgical complexity is an important factor affecting the type and severity of infection after spinal surgery.^[2]^ (Fig. 1) Our study was limited to surgeries involving arthrodesis and fusion; simple spine surgeries were not included. In contrast, the NNIS report included data from general spine surgeries, which include simple spine surgeries. Therefore, the rate of SSI in our study was expected to be higher than that reported for general spine surgery, because spinal fusion surgery involves a longer operation time and more complicated technique than simple spine surgeries. Another factor that can affect infection rates is the strict application of the CDC criteria. There are 4 items per criterion, and a criterion is fulfilled if 1 or more items are satisfied. In our study, we applied the infection standard as widely as possible because our goal was to identify factors that affected the infection rate, rather than the overall infection rate. The high overall infection rate identified in this study was therefore partially attributed to this broad application of the criteria.

### 4.2. Importance of SFA expertise

Various authors have documented the importance of surgical team expertise as a procedure-related risk factor associated with postoperative SSI.^[1]^ Undoubtedly, the surgical experience of the operating surgeon is the most important factor in determining the expertise of the surgical team. However, surgeons are unable to perform surgeries alone and require an operating team that includes at least 1 assistant. The SFA has several roles, ranging from providing aid to the operating surgeon to acting as the operating surgeon when the surgeon leaves the operative field after a major procedure. Therefore, it is necessary to consider the effect of the SFA on the results of the operation, including postoperative complications, such as SSI.^[23]^ In 2012, the perioperative care collaborative defined an SFA as a qualified practitioner who provides continuous, competent, and dedicated assistance under the direct supervision of the operating surgeon throughout the procedure, and published a review of the SFA role.^[24]^ Subsequently, Quick reaffirmed the position statement issued by the perioperative care collaborative and clarified the role of the SFA.^[25]^ Kim et al^[23]^ evaluated the impact of a novice SFA on operative outcomes during laparoscopic colorectal surgery and found that novice SFAs were associated with significantly increased operative times, although the rate of overall postoperative complications was not significantly higher. See et al^[26]^ concluded that the incidence of postoperative complications increases in surgeries involving inexperienced trainee assistants such as fellows, residents, and interns, who are rotated continuously for training purposes. A review by Drake et al^[27]^ revealed a decrease in the chief resident operative volume and narrowed operative experience. Therefore, it is illogical to expect the resident trainee to perform the SFA role perfectly. Suzzane et al reported the importance of SFA expertise in Coronary Artery Bypass Grafting surgery.^[28]^ The study compared the rates of leg harvest SSIs after surgeries in which advanced nurse practitioners served as the SFA to those of leg harvest SSIs after surgeries in which less experienced surgical residents served as the SFA. Patients undergoing cardiac surgery in which the SFA was an advanced nurse practitioner were less likely to develop leg harvest SSI and had shorter operative times. In our study, an attending physician, rather than an advanced nurse practitioner, served as the SFA in the high-expertise group, although similar results were observed. This outcome is not surprising, considering that advanced nurse practitioners and attending physicians have similar levels of expertise. Based on the above reports and our findings, SFA expertise is significantly associated with improved outcomes in surgery, including reduced SSI rates

In conclusion, our study provides the first analysis of the effect of SFA expertise on SSI incidence following instrumented spinal surgery. These findings will serve as the foundation for developing future strategies to minimize SSI risk, and consequently, patient morbidity, death, and healthcare expenses.

## Author contributions

**Conceptualization:** Deok Ryeong Kim, Byung Gwan Moon.

**Data curation:** Deok Ryeong Kim, Byul Hee Yoon.

**Formal analysis:** Deok Ryeong Kim, Yung Ki Park.

**Investigation:** Deok Ryeong Kim.

**Methodology:** Deok Ryeong Kim, Byul Hee Yoon.

**Software:** Byul Hee Yoon, Yung Ki Park.

**Writing – original draft:** Deok ryeong Kim.

**Writing – review & editing:** Byung Gwan Moon, Byul Hee Yoon, Yung Ki Park.
